# Semaphorins or Frizzled –it is the receptor that direct the action of clostridial glucosylating toxins

**DOI:** 10.1038/s41392-020-00307-3

**Published:** 2020-09-19

**Authors:** Klaus Aktories

**Affiliations:** Institute of Experimental and Clinical Pharmacology and Toxicology, Medical Faculty, University of Freiburg, Albertstr. 25, 79104 Freiburg, Germany

**Keywords:** Cell biology, Microbiology

In a recent article in *Cell*, Lee et al.^1^ establish semaphorins SEMA6A and SEMA6B as physiologically relevant receptors for *Paeniclostridium sordellii* lethal toxin (TcsL) which can cause lethal toxic shock syndrome and reveal the molecular basis for the difference in tissue targeting and disease pathogenesis between highly related toxins TcsL and *C. difficile* TcdB.

Large clostridial glucosylating toxins (Lgts) are major virulence factors crucially involved in different types of diseases. Prototypes are toxin A (TcdA) and toxin B (TcdB) from *Clostridioides difficile* (formerly *Clostridium difficile*), which are responsible for *C. difficile*-induced antibiotic-associated diarrhea and pseudomembranous colitis.^2^ The related toxin TcsL is produced by *Paeniclostridium sordellii* (formerly termed *Clostridium sordellii*), a pathogen that in humans causes edema, gangrene and myonecrosis often followed by fatal toxic shock syndrome; in animals *P. sordellii* infections are more frequent, leading to large outbreaks of enterotoxemia.^3^ TcsL is extremely toxic surpassed only by *C. botulinum* and *C. tetani* neurotoxins.^3^ Notably, TcsL is the nearest relative of TcdB, showing 76% sequence identity (sequence identity of TcsL with *C. difficile* TcdA is only 43%). Both toxins share the same structural architecture (Fig. 1a), consisting of the N-terminal glucosyltransferase, which is followed by an auto-protease domain, a delivery domain and a so-called CROP domain. All Lgts inactivate Ras/Rho switch proteins by glucosylation. Main substrates of TcsL are Rac and Ras, while TcdB prefers Rho and Rac.^2^

**Fig. 1:**
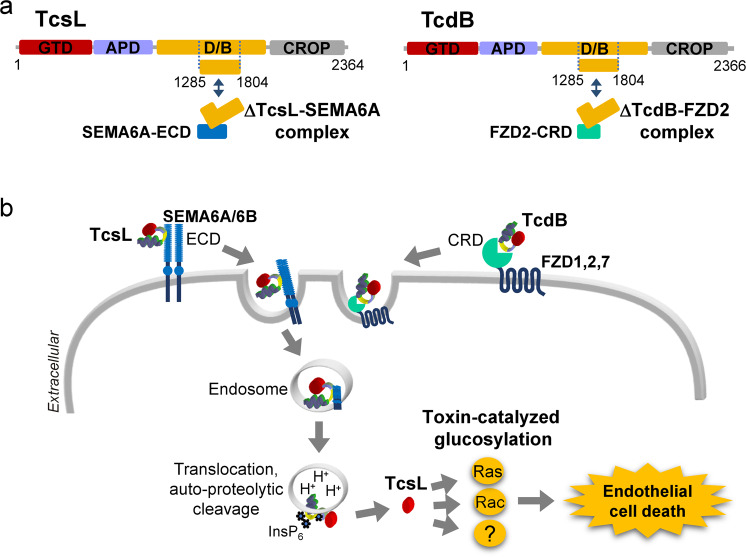
Interaction of TcsL with semaphorins. **a** Schemes of the structures of TcsL and TcdB. Both toxins consist of the glucosyltransferase domain (GTD), the auto-protease domain (APD), the delivery and binding domain (D/B), and the C-terminal CROP (combined repetitive oligopeptide) domain. For Cryo-EM studies of the interaction of TcsL with the extracellular domain of Semaphorin 6A, a TcsL region located in D/B (residues 1285–1804) was used. Exactly this region was employed for the crystal structure analysis of TcdB in complex with the cysteine-rich domain (CRD) of Frizzled 2 (FZD2). The interaction of both toxins with their specific receptors is almost identical. **b** Scheme of the action of TcsL. TcsL binds to the extracellular domain (ECD) of Sema6A and 6B (which form dimers) within the D/B domain. Then, the toxin receptor complex is endocytosed. At low pH of endosomes, the toxin inserts into endosomal membranes and translocates the GTD and APD domains into the cytosol. Here, APD is activated by inositol hexakisphosphate (InsP_6_) and releases GTD. TcsL GTD glucosylates Rac and Ras proteins (and probably other related GTPases), thereby causing destruction of the cytoskeleton and cell death. The interaction of TcdB with its major receptor Frizzled (FZD1, 2, 7) is indicated. Further uptake steps of TcdB (probably similar to TcsL) are not shown

The identification of the cell surface receptor(s) is crucial for the understanding of the pathogenesis of these toxins. Recently, several host cell receptors for TcdB have been identified, including Frizzled proteins 1, 2, and 7, which are heptahelical G-protein-coupled receptors involved in Wnt signaling. Now, two most exciting, independent studies identified the host receptor for lethal toxin TcsL.^1,4^ Both research groups show that TcsL interacts with semaphorin 6A and 6B to intoxicate host cells. About 20 semaphorins exist, which belong to at least eight different families (two in invertebrates, five in vertebrates and one in virus). They are cell membrane-bound or soluble factors and are characterized by the N-terminal Sema domain (with a seven-blade β-propeller fold) followed by a cysteine-rich region (called plexin-semaphorin-integrin (PSI) domain). Initially, semaphorins were recognized as repellent cues for axon guidance; now, it is clear that diverse cellular functions are regulated by these proteins, including immune regulation, cell proliferation and angiogenesis. Physiological receptors of semaphorins are plexins and neuropilins. However, additional receptors are suggested.

Using CRISPR/Cas9 screening, both research groups identified Semaphorin 6A as receptor for TcsL. In addition, SEMA6B, which is 53% identical with SEMA6A, but not SEMA6C or SEMA6D, serves as a TcsL receptor.^1,4^ The sensitivity of cells towards TcsL is directly related to the amount of expressed SEMA proteins.^1^ Moreover, the extracellular domain (ECD) of SEMA6A protects cells against intoxication, when TcsL is pre-incubated with ECD or when ECD is immediately (up to 5 min) added after the toxin.^1,4^ Because vascular endothelium of the lung appears to be the primary target of TcsL,^3^ Lee and co-workers used human lung microvascular cells (HULECs),^1^ which express high amounts of SEMA6A and 6B receptors. These cells show 50% growth inhibition at 50 femtomolar concentrations (200 molecules per cell are lethal). Importantly, both research groups show strong protective effects of semaphorin ECD in mice, and, especially, lung edema formation was largely reduced.^1,4^

Lee et al.^1^ succeeded in obtaining the Cryo-EM structure of SEMA6A with the TcsL fragment, covering residues 1285 through 1804. They chose this region because an analogous fragment of TcdB was recently successfully used for crystal structure analysis of the interacting TcdB-Frizzled2 complex,^5^ which is located in the delivery domain of the toxin (Fig. 1a). Starting with a glutaraldehyde cross-linking approach, they succeeded in a local resolution up to 2.8 Å at the binding interface. They show that TcsL binds SEMA6A similarly as Plexin A2, which is its physiological receptor. Accordingly, Plexin A2 interacts with SEMA6A and SEMA6B but not with SEMA6C and 6D. Moreover, a critical amino acid Ile193, which is conserved in SEMA6A and SEMA6B (but not in SEMA6C and 6D isoforms) appears to be crucial for binding to TcsL and Plexin A2. More excitingly, the interaction of TcsL with semaphorins occurs in a convex region of the delivery domain of the toxin, formed by an antiparallel beta sheet (residues 1466–1511) flanked by an α-helix (1433–1438) and a loop (1596–1511). Exactly this region (termed *vertex of the ‘L’ shaped* TcdB-FZD binding domain in ref. ^5^) is used in TcdB for interaction with its receptor FZD2. Exchange of the proposed crucial amino acids involved in the interaction of TcsL with SEMA6A verified the interaction site. Moreover, the interaction of TcdB with FZD2 is characterized by a palmitoleic bridge buried in a hydrophobic pocket of TcdB.^5^ The respective hydrophobic pocket in TcsL is filled by the hydrophobic side chain of methionine 109 of SEMA6A.^1^ Remarkably, the direct interacting regions of TcsL with SEMA6A and of TcdB with FZDs are largely different in both toxins although the overall identity of TcsL and TcdB is 76%, indicating that divergency developed specifically for the different receptors. Notably, when 15 TcsL residues were changed to those of TcdB, the binding of TcsL was switched from SEMA6A to FZD.^1^ Nicely, the work by the group around Min Dong came to similar conclusions about the interaction region using TcdB/TcsL chimeras.^4^ These results support a specific role of the “delivery domain” of Lgts in receptor interaction. For TcdB, at least two other cell surface receptors (e.g., chondroitin sulfate proteoglycan 4 (CSPG4) and poliovirus receptor-like3 (PVRL3)) have been identified. The authors of both papers emphasize that they could not exclude that additional TcsL receptors exist; however, the *in vivo* studies indicate that SEMA6A/B is the pathophysiological relevant receptor. Again, this study shows the power of CRISPR screening for identification of toxin receptors, which may be an important prerequisite for targeted therapies. Last not least, worth mentioning that Tian et al. also identified TBC1D3, which is a GTPase activating protein (GAP) for Rab5 proteins, in their CRISP-Cas9 screen.^4^ This GAP did not affect TcdB toxicity. What is then its action in TcsL toxicity? Hopefully, we will get the answer soon.

## Funding

Open access funding provided by Projekt DEAL.
